# Establishing Crosswalks Between Common Measures of Burnout in US Physicians

**DOI:** 10.1007/s11606-021-06661-4

**Published:** 2021-03-31

**Authors:** Keri J. S. Brady, Pengsheng Ni, Lindsey Carlasare, Tait D. Shanafelt, Christine A. Sinsky, Mark Linzer, Martin Stillman, Mickey T. Trockel

**Affiliations:** 1 Health Law, Policy & Management Department, Boston University School of Public Health, Boston, MA USA; 2 Biostatistics & Epidemiology Data Analytic Center, Boston University School of Public Health, Boston, MA USA; 3 American Medical Association, Chicago, IL USA; 4 Stanford Medicine WellMD Center, Stanford University, Stanford, CA USA; 5 Hennepin Healthcare Research Institute and Department of Medicine, Hennepin Healthcare, University of Minnesota, Minneapolis, MN USA; 6 Department of Psychiatry and Behavioral Sciences, Stanford University, Stanford, CA USA

**Keywords:** physician burnout, physician well-being, burnout measurement

## Abstract

**Background:**

Physician burnout is often assessed by healthcare organizations. Yet, scores from different burnout measures cannot currently be directly compared, limiting the interpretation of results across organizations or studies.

**Objective:**

To link common measures of burnout to a single metric in psychometric analyses such that group-level scores from different assessments can be compared.

**Design:**

Cross-sectional survey.

**Setting:**

US practices.

**Participants:**

A total of 1355 physicians sampled from the American Medical Association Physician Masterfile.

**Main Measures:**

We linked the Stanford Professional Fulfillment Index (PFI) and Mini-Z Single-Item Burnout (MZSIB) scale to the Maslach Burnout Inventory (MBI) in item response theory (IRT) fixed-calibration and equipercentile analyses and created crosswalks mapping PFI and MZSIB scores to corresponding MBI scores. We evaluated the accuracy of the results by comparing physicians’ actual MBI scores to those predicted by linking and described the closest cut-point equivalencies across scales linked to the same MBI subscale using the resulting crosswalks.

**Key Results:**

IRT linking produced the most accurate results and was used to create crosswalks mapping (1) PFI Work Exhaustion (PFI-WE) and MZSIB scores to MBI Emotional Exhaustion (MBI-EE) scores and (2) PFI Interpersonal Disengagement (PFI-ID) scores to MBI Depersonalization (MBI-DP) scores. The commonly used MBI-EE raw score cut-point of ≥27 corresponded most closely with respective PFI-WE and MZSIB raw score cut-points of ≥7 and ≥3. The commonly used MBI-DP raw score cut-point of ≥10 corresponded most closely with a PFI-ID raw score cut-point of ≥9.

**Conclusions:**

Our findings allow healthcare organizations using the PFI or MZSIB to compare group-level scores to historical, regional, or national MBI scores (and vice-versa).

**Supplementary Information:**

The online version contains supplementary material available at 10.1007/s11606-021-06661-4.

## INTRODUCTION

In the US, burnout is more common in physicians than in workers in other fields,^1^ and is characterized by work-related feelings of exhaustion and depersonalization or interpersonal disengagement.^2, 3^ Physician burnout is associated with poor physician health outcomes, reduced quality of care, and at least 4.6 billion dollars in excess health system costs annually.^4–6^ In an effort to curb physician burnout,^7, 8^ health systems across the nation are integrating measures of burnout into routine organizational assessments to monitor system functioning and evaluate the effectiveness of practice changes designed to improve physician well-being.^9–11^ This practice is recommended in the National Academy of Medicine’s consensus report on clinician burnout and regarded by healthcare leaders as a basic first step to addressing the problem.^7, 10–14^

With the widespread adoption of physician burnout assessment within US healthcare systems has come the problem of comparing outcomes across different burnout measures. With several validated options available that vary in length and cost, a number of different measures are currently in use in the US,^9, 10^ including the Maslach Burnout Inventory-Human Services Survey for Medical Personnel (MBI),^15^ Stanford Professional Fulfillment Index (PFI),^16^ and the Mini-Z Single-Item Burnout (MZSIB) scale.^17^ When two different burnout measures are used across organizations or within an organization over time, the scores are not comparable unless they are placed onto the same metric, or “linked,” in psychometric analyses. To date, no studies to our knowledge have linked common measures of physician burnout onto a single metric, which would allow healthcare organizations to compare burnout scores/rates across different measures.

The primary aim of this study was to link the PFI and MZSIB to the MBI metric and create crosswalks that map scores from the PFI and MZSIB to corresponding scores on the MBI. Using the crosswalks, we aimed to describe the closest cut-point equivalences for scales linked to the same metric. Our secondary aim was to examine the psychometric properties of scales linked to the same metric, including each scale’s reliability and associations with relevant adverse outcomes.

## METHODS

Linking refers to the statistical process of placing two or more measures with different content and/or construct severity levels onto the same scale.^18^ Through this process, a relationship is established between the linked measures, such that for each score on Burnout Measure A, an equivalent score (within standard error) on Burnout Measure B is established.

### Design and Participants

This study used a single-group linking design, whereby items from each burnout instrument were administered in a confidential, cross-sectional survey to all respondents from February to March 2019. To obtain a representative convenience sample, we randomly sampled physicians of all ages, sexes, and specialties from the American Medical Association Physician Masterfile. Physicians were emailed the survey and offered a small financial incentive to participate. The survey was administered in waves until we reached a target sample size of ≥1200 respondents, which was estimated as the minimum sample size needed for item response theory linking analyses. Physicians (including postgraduate trainees) practicing in the US at the time of the survey were eligible for inclusion.

### Measures

We measured physician burnout using the MBI 9-item Emotional Exhaustion (MBI-EE) and 5-item Depersonalization (MBI-DP) subscales (0 = never, 1 = a few times a year or less, 2 = once a month or less, 3 = a few times a month, 4 = once a week, 5 = a few times a week, 6 = every day); the PFI 4-item Work Exhaustion (PFI-WE) and 6-item Interpersonal Disengagement (PFI-ID) subscales (0 = not at all, 1 = very little, 2 = moderately, 3 = a lot, 4 = extremely); and the single-item MZSIB (1 = no burnout; 2 = under stress; 3 = have one or more burnout symptom; 4 = burnout won’t go away; 5 = completely burned out; see Supplemental Appendix 1 for the complete MZSIB response options).^17^ The sequence in which each instrument was administered was randomized to prevent ordering effects.

The MBI and PFI are outcome measures, whereas the MZSIB scale is a screening measure. Commonly used raw (total) score cut-points for each scale are ≥27, ≥10, and ≥3 on the MBI-EE, MBI-DP, and MZSIB scales, respectively.^1, 19, 20^ The raw (total) score cut-point for the PFI Burnout Composite (PFI-BC) Scale is ≥14.^16^ Cut-points for PFI-WE and PFI-ID subscales have not been published and are identified in the current study.^16^

We also assessed physicians’ demographics, depressive symptoms (4-item PROMIS depression measure),^21^ distress as measured by the original, 7-item Physician Well-Being Index (WBI),^22–24^ and intent to leave one’s current practice or intent to leave medicine (for attending physicians and postgraduate trainees, respectively) in the next 2 years (1 item). ^17^ All measures were scored such that higher scores indicate more of each construct.

### Linking Analyses

Our methods were informed by those used in the PROsetta Stone Project.^25, 26^ Scales were linked in item sets, consisting of two scales: a *target* measure and an *anchor* measure. In linking analyses, a target measure is linked *to* an anchor measure, which places the target measure onto the metric of the anchor measure. Because the MBI is historically the most common physician burnout assessment,^27^ we selected the MBI-EE and MBI-DP scales as anchor measures. Target measures included the PFI-WE, PFI-ID, and MZSIB scales.

Prior to conducting linking analyses, we qualitatively and quantitatively examined the degree to which the scales that we aimed to link assess essentially the same construct, a key assumption of linking.^18, 28^ Scales assessing essentially the same construct were expected to (1) have very similar item content as determined by two independent subject domain expert raters (TS, ML); (2) be highly correlated (inter-scale Pearson’s *r* of ≥0.75); and (3) be essentially unidimensional as determined in confirmatory factor analyses (CFAs) (see Supplemental Appendix 2 for additional assumption assessment details).^25^

For each item set, we conducted item response theory (IRT) fixed-calibration linking and equipercentile linking analyses using a fivefold cross validation process (Supplemental Appendix 3). In IRT linking, raw (total) scores on each target measure were linked to t-scores on each MBI anchor scale. A t-score is a standardized score ranging from 0 to 100, with a mean score and standard deviation equal to 50 and 10, respectively. T-scores on each MBI anchor scale were then mapped to corresponding MBI raw scores. In our IRT linking analyses, we derived the MBI-EE and MBI-DP anchor metrics from a prior IRT calibration of the MBI in a 2014 national sample of US physicians.^29, 30^ In equipercentile linking, the MBI metric was derived from the primary survey data collected in this study. We evaluated the accuracy of each linking method for each item set by calculating the correlation, mean difference, and standard deviation (SD) of the difference between physicians’ predicted and actual t-scores on the MBI anchor scale, using pooled predicted and actual t-scores produced from a fivefold cross validation process. The method that yielded the highest correlations, lowest mean differences, and lowest SD of difference across all item sets was used to create a crosswalk mapping raw scores on the target measure to corresponding t-scores and raw scores on the MBI anchor measure. Once each item set was linked, we (1) identified the closest cut-point equivalencies across scales linked to the same metric and (2) described the reliability of scales linked to the same metric (Supplemental Appendix 4).^31^ We used the Brady et al. ^29^ IRT analysis to identify the t-scores corresponding with (1) each MBI-EE and MBI-DP raw score cut-point and (2) each raw score on the MBI predicted by equipercentile linking.

Finally, we computed correlations between each scale and measures of physician depressive symptoms, distress, and intent to leave to compare the magnitude of each scale’s associations with these outcomes. Analyses were conducted in R (v3.5.1) *psych*, *lavaan*, *mirt*, and *equate* packages.^32–36^ This study was approved by the University of Illinois at Chicago Institutional Review Board.

## RESULTS

### Sample

The overall sample included 1355 US physicians (Table 1). The most common demographic characteristics of respondents were White race, male sex, non-primary care specialty, and <44 years of age. Thirty-one percent of respondents were trainees. In subgroup invariance analyses, we found support for the invariance of our linking results across early versus late responders (where late responders were used as a proxy for non-responders; Supplemental Appendix 5, Table 5.5). Overall, mean raw scores on the MBI-EE, PFI-WE, MZSIB, MBI-DP, and PFI-ID scales were 21.82, 6.06, 2.45, 7.86, and 6.63, respectively (Table 2) (see Supplemental Appendix 6 for specialty-level descriptive scale statistics).

**Table 1:** Overall Sample Characteristics (*n* = 1355)

Characteristic	*n* (%)^a^
Sex	
Male	763 (57)
Female	579 (43)
Missing	13 (0.1)
Age group	
<35 years	440 (33)
35–44 years	385 (28)
45–54 years	243 (18)
55–64 years	193 (14)
***≥***65 years	94 (7)
Race	
White/Caucasian	894 (66)
Black/African American	54 (4)
Asian	292 (22)
Other	115 (9)
Trainee status	
Trainee (resident/fellow)	420 (31)
Non-trainee	935 (69)
Primary care	
Primary care^b^	442 (33)
Non-primary care	913 (67)
Specialty	
Anesthesiology	97 (7)
Dermatology	24 (2)
Emergency medicine	74 (6)
Family medicine	167 (12)
General surgery	62 (5)
General surgery subspecialty	71 (5)
General internal medicine	184 (14)
General pediatrics	91 (7)
Internal medicine-subspecialty	127 (9)
Neurology	28 (2)
Obstetrics and gynecology	96 (7)
Ophthalmology	30 (2)
Other	81 (6)
Pathology	4 (0.3)
Pediatric subspecialty	63 (5)
Physical medicine	13 (1)
Psychiatry	90 (7)
Radiology	53 (4)
Practice type	
Non-governmental hospital	473 (35)
Group practice	404 (30)
City/county/state/federal government hospital	130 (10)
Self-employed solo practice	97 (7)
Other^c^	250 (18)
Missing	1 (0.1)

^a^Percentages may not add to 100 due to rounding. Missingness is only specified for variables that had missing data. ^b^Includes physicians in general internal medicine, pediatrics, and family medicine specialties. ^c^Includes physicians practicing in/as HMO, locum tenens, medical school, two physician practice (full or part owner), other patient care, city/county/state government non-hospital setting, and no classification

**Table 2:** Overall Descriptive Scale Statistics by Domain and Measure (*n* = 1346)

Domain/measure	Statistic^a^
Emotional exhaustion	
MBI-EE, mean (SD)	21.82 (12.16)
MBI-EE ≥27, *n* (%)	469 (34.8)
PFI-WE, mean (SD)	6.06 (3.46)
PFI-WE ≥7, *n* (%)	582 (43.2)
MZSIB, mean (SD)	2.45 (0.92)
MZSIB ≥3, *n* (%)	589 (43.8)
Depersonalization	
MBI-DP, mean (SD)	7.86 (6.41)
MBI-DP ≥10, *n* (%)	458 (34.0)
PFI-ID, mean (SD)	6.63 (4.77)
PFI-ID ≥9, *n* (%)	470 (34.9)
Burnout	
MBI (EE ≥27 and/or DP ≥10), *n* (%)	584 (43.4)
PFI-BC^b^, mean (SD)^b^	12.68 (7.63)
PFI-BC^b^ ≥14, *n* (%)^b^	599 (44.5)

^a^Includes respondents with ≤1 missing item response for all scales. Cut-points presented are raw total scores on each scale. ^b^PFI BC refers to the PFI Burnout Composite Scale, which is scored as the total raw score from both the PFI-WE and PFI-ID scales

### Assumption Assessment

In qualitative evaluations of each target and anchor scale’s item content overlap, both raters agreed that the following item sets assess essentially the same underlying construct: PFI-WE and MBI-EE (item set 1), PFI-ID and MBI-DP (item set 2), and MZSIB and MBI-EE (item set 3). Inter-scale correlations between the target and anchor scales in item sets 1–3 were 0.80, 0.76, and 0.76, respectively. Item sets 1–3 met all other linking assumptions in quantitative analyses (Supplemental Appendix 5).

### Crosswalks and Closest Cut-Point Equivalents

Overall, IRT (versus equipercentile) linking produced the most accurate results (Supplemental Appendices 7 - 9) and was used to create crosswalks mapping raw scores on the PFI-WE, PFI-ID, and MZSIB (target) scales to corresponding t-scores and raw scores on their respective MBI-EE, MBI-DP, and MBI-EE anchor scales (Table 3).

The commonly used raw score cut-point of ≥27 (t-score = 50.70) ^29^ on the MBI-EE scale corresponded most closely with raw score cut-points of ≥7 and ≥3 on the respective PFI-WE and MZSIB scales (Table 3). The commonly used raw score cut-point of ≥10 (t-score = 53.76) ^29^ on the MBI-DP scale corresponded most closely with a raw score cut-point of ≥9 on the PFI-ID scale. The raw score cut-point of ≥3 on the MZSIB scale corresponded most closely with a raw score of ≥8 on the PFI-WE scale.

**Table 3:** Crosswalks Produced from IRT Linking Mapping Raw Scores from the PFI and MZSIB to Corresponding Predicted MBI T-scores and Raw Scores

Item Set 1: PFI Work Exhaustion (PFI-WE) Scale (target scale) linked to MBI Emotional Exhaustion (MBI-EE) Scale (anchor scale)^a^			Item Set 2: PFI Interpersonal Disengagement (PFI-ID) Scale (target scale) linked to MBI Depersonalization (MBI-DP) Scale (anchor scale)^a^			Item Set 3: Mini-Z Single-Item Burnout (MZSIB) Scale (target scale) linked to MBI Emotional Exhaustion (MBI-EE) Scale (anchor scale)^a^		
PFI-WE raw (total) score	Predicted MBI-EE T-score (SE)	Predicted MBI-EE raw (total) score	PFI-ID Scale raw (total) score	Predicted MBI-DP Scale T-score (SE)	Predicted MBI-DP raw (total) score	MZSIB item raw score	Predicted MBI-EE Scale T-score (SE)	Predicted MBI-EE raw score
0	30.15 (4.93)	2.57	0	35.46 (5.36)	1.31	1	35.44 (6.26)	6.27
1	34.96 (3.79)	5.86	1	40.99 (3.59)	2.41	2	44.75 (5.35)	17.64
2	38.09 (3.52)	8.89	2	42.76 (3.58)	2.99	**3**	**52.37 (5.05)**	**29.92**
3	40.74 (3.39)	12.06	3	45.06 (3.18)	3.94	4	60.09 (5.60)	40.62
4	43.10 (3.33)	15.24	4	46.90 (3.21)	4.85	5	69.49 (6.30)	48.80
5	45.34 (3.32)	18.54	5	48.51 (3.10)	5.74			
6	47.59 (3.32)	22.09	**6**	**50.19 (3.02)**	**6.77**			
**7**	**49.81 (3.30)**	**25.73**	7	51.81 (3.07)	7.89			
8	51.98 (3.29)	29.29	8	53.30 (3.10)	9.01			
9	54.13 (3.30)	32.65	9	54.91 (3.07)	10.30			
10	56.33 (3.31)	35.80	10	56.47 (3.11)	11.62			
11	58.55 (3.31)	38.72	11	57.93 (3.07)	12.88			
12	60.77 (3.32)	41.43	12	59.47 (3.04)	14.22			
13	63.11 (3.38)	44.04	13	60.92 (3.10)	15.47			
14	65.71 (3.49)	46.41	14	62.31 (3.10)	16.64			
15	68.75 (3.75)	48.41	15	63.85 (3.08)	17.87			
16	73.18 (4.70)	50.36	16	65.35 (3.09)	19.01			
			17	66.84 (3.02)	20.07			
			18	68.40 (2.98)	21.11			
			19	69.95 (3.02)	22.09			
			20	71.52 (3.05)	23.01			
			21	73.28 (3.03)	23.94			
			22	75.24 (3.16)	24.83			
			23	77.17 (3.21)	25.58			
			24	80.61 (3.98)	26.69			

^a^Bolded values are those that are closest to the mean on the corresponding MBI anchor metric. Crosswalks were generated using item response theory fixed-calibration linking based on MBI item parameter estimates established in prior IRT analysis of MBI data from a 2014 national physician sample.^29^ Note that item set 2 is not on the same metric as item sets 1 and 3. Therefore, item set 2 cannot be compared with item sets 1 and 3

### Reliability

**Figure 1:**
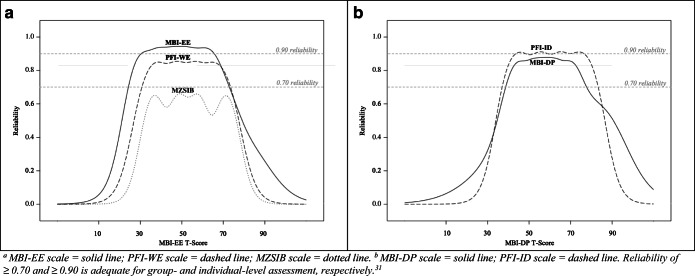
**Scale Reliability Across the MBI Anchor Scale T-score Metrics for Scales Linked to the Same Anchor Metric. a Reliability of the MBI-EE, PFI-WE, and MZSIB scales across MBI-EE t-score metric (MBI-EE scale,** ***solid line*; PFI-WE scale,** ***dashed line*; MZSIB scale,** ***dotted line*). Figure 1b; b Reliability of the MBI-DP and PFI-ID scales across MBI-DP t-score metric (MBI-DP scale,** ***solid line*; PFI-ID scale,** ***dashed line*. Reliability of ≥0.70 and ≥0.90 is adequate for group- and individual-level assessment, respectively**^31^**)**.

Both the MBI-EE and PFI-WE scales demonstrated ≥0.70 reliability to assess a wide range of low and high emotional exhaustion levels on the MBI-EE t-score metric (Fig. 1a). The MZSIB scale showed less than 0.70 reliability to assess emotional exhaustion across the MBI-EE t-score metric. Both the MBI-DP and PFI-ID scales also demonstrated ≥0.70 reliability to assess a range of low and high depersonalization levels on the MBI-DP t-score metric (Fig. 1b). Compared to the PFI-WE scale, the MBI-EE scale possessed ≥0.70 reliability over a wider range of below average emotional exhaustion t-scores, whereas, compared to the MBI-DP scale, the PFI-ID scale possessed ≥0.70 reliability over a wider range of above average depersonalization t-scores.

### Associations with Adverse Outcomes

All scales correlated with physician depressive symptoms, physician distress, and physicians’ intent to leave their practice or medicine within 2 years (Table 4). Among measures assessing the same underlying construct (i.e., the MBI-EE, PFI-WE, and MZSIB measures of emotional exhaustion and the MBI-DP and PFI-ID measures of depersonalization), there were no major differences in the magnitude of correlations between each burnout scale and depressive symptom, distress, and intent to leave outcomes (Table 4). The MBI-DP scale showed a modestly lesser correlation with intent to leave compared to the PFI-ID scale.

**Table 4:** Correlation Analysis of Each Scale’s Raw Scores with Adverse Outcomes

Outcome	Emotional Exhaustion Measures^a^			Depersonalization Measures^a^	
	MBI-EE	PFI-WE	MZSIB	MBI-DP	PFI-ID
Depressive symptoms	0.63	0.64	0.59	0.53	0.54
Distress^b^	0.71	0.71	0.70	0.60	0.60
Intent to leave one’s practice (attending) or medicine (trainee) in two years	0.18	0.21	0.21	0.14	0.20

^a^All correlations are Spearman correlations; all correlations are significant at *p* < 0.05; ^b^ defined by burnout, depression, mental quality of life, physical quality of life, stress, and fatigue

## DISCUSSION

Healthcare organizations across the US are monitoring physician burnout as an indicator of health system performance.^9^ Common applications of physician burnout measurement as a performance indicator are to make inferences regarding the quality of physicians’ medical practice environments, workforce sustainability, and healthcare quality.^9^ Yet, comparisons of performance over time, across organizations, or across studies are not possible when different burnout measures have been employed. In this study, we used IRT linking to place common burnout measures—the PFI and MZSIB—onto the metric of the MBI, and created crosswalks that map raw scores on the PFI-WE, PFI-ID, and MZSIB scales to corresponding MBI subscale scores. For scales linked to the same metric, we identified the closest cut-point equivalencies across all linked metrics and compared the reliability across linked outcome metrics.

By linking the PFI, MZSIB, and MBI to the same metric, the crosswalks we produced allow investigators using these measures to make several useful comparisons.^25^ First, investigators can compare summary sample scores across the PFI, MZSIB, and MBI. That is, using the crosswalks produced in this study, group-level emotional exhaustion scores can be compared across the MBI-EE, PFI-WE, and MZSIB scales, and group-level depersonalization scores can be compared across the MBI-DP or PFI-ID scales.^25^ Second, investigators can use the crosswalks to calculate emotional exhaustion/depersonalization rates across metrics by substituting respondents’ raw (total) scores on the PFI or MZSIB with the corresponding MBI t-score. The corresponding MBI t-scores can then be used to calculate the percent of physicians scoring at or above a selected MBI cut-point. The substituted MBI scores can be further analyzed in descriptive and inferential analyses.^25^ The crosswalks can also be used to calculate emotional exhaustion/depersonalization rates across metrics using only aggregated data. In Supplemental Appendix 10, we demonstrate how to calculate emotional exhaustion/depersonalization rates on the MBI metric using frequency tables of physicians’ raw scores on the PFI. The crosswalks can facilitate comparisons of burnout scores/rates across organizations using different measures, within organizations using different measures over time, and to published regional/national benchmarks. The use of our crosswalks to convert burnout scores from different measures to a common metric may also improve comparative effectiveness and meta-analysis research by reducing error associated with the use of different scales across studies.^25, 37^

Our reliability assessment provides important information regarding the psychometric performance of each measure, each of which has its own strengths and weaknesses that should be considered within the intended purpose of an organization’s assessment.^9^ For example, the MBI-EE scale provides >0.90 reliability to assess a wide range of emotional exhaustion levels, but at the cost of additional items. With less than half the items of the MBI-EE, the PFI-WE scale offers >0.80 reliability to assess a similar range of above-average emotional exhaustion levels as the MBI-EE scale, but has less precision at below average emotional exhaustion levels than the MBI-EE scale. Similarly, with only one item, the MZSIB offers the least response burden but has less precision to assess emotional exhaustion than the MBI-EE and PFI-WE scales (an expected result given the MZSIB was originally designed as a brief screening tool, not an outcome assessment). However, this level of precision may be sufficient, for example, if the intended purpose of assessment is for screening followed by additional assessment, or to predict the risk of occupational outcomes of depression symptoms, distress, or intent to leave one’s practice at a group-level. The PFI-ID scale offers the most reliable assessment of depersonalization across the widest range of depersonalization levels, with one additional item compared to MBI-DP scale. We should note that, to our knowledge, this is the first assessment of the MZSIB’s reliability (as internal consistency reliability is not applicable to single-item scales and test-retest reliability has not yet been investigated for this measure).

All scales showed significant correlations with important, adverse outcomes, including physician depression, distress, and intent to leave. The association between each measure and each adverse outcome underscores the importance of including measures of physician burnout in institutional assessments.

To our knowledge, this is the first study to crosswalk common measures of burnout among US physicians. Strengths of this study include the use of a single-group linking design (permitting the direct comparison of physicians’ actual MBI scores to those predicted by linking to determine the accuracy of our results) and the use and agreement of two different linking methods.

However, this study has several limitations. First, because the MBI-EE and MBI-DP metrics to which the PFI and MZSIB are linked were derived from a prior IRT analysis of 2014 MBI data from the Shanafelt et al. (2015) national physician burnout prevalence study,^29, 30^ the mean of each MBI anchor scale is fixed to the mean EE and DP scores of US physicians in 2014. Therefore, when interpreting a score on a target scale relative to its SDs above/below the mean score on its MBI anchor scale, it should be known that the comparison is relative to the underlying mean MBI score of US physicians in 2014. Despite this limitation, the crosswalks remain valid assuming that the MBI subscales function equivalently across the 2014 US physician sample and US general physician population. Second, although our findings provide support for the invariance of our crosswalks across early and late responder groups and, therefore, provide potential support for the representativeness of our sample, this support relies on the assumption that late responders are an adequate proxy for non-respondents. Nevertheless, several studies have demonstrated no significant differences in burnout estimates across respondent and non-respondent groups, despite the low response rates that are common in physician survey research.^1, 38^ Third, we chose to highlight the closest cut-point equivalencies across linked measures using commonly used cut-points on each metric. Because raw scores on each target metric are linked to continuous scores on each anchor metric, the closest cut-point equivalencies across metrics are an approximation. Although we identified the closest cut-point equivalency for scores ≥27 and ≥10 on the respective MBI-EE and MBI-DP scales, investigators can use crosswalks published in Brady et al. ^29^ in conjunction with the crosswalks presented herein to identify cut-point equivalencies on the PFI and MZSIB at other MBI raw score cut-points.

It is important to note that the crosswalk tables rendered with this research allow reasonable approximate translation of *aggregate, group-level* scores from one measure of burnout to another. They are not intended to translate individual-level respondent scores from one measure of burnout to another, and attempting to do so would produce unreliable results. In addition, it is important to note that crosswalking scores from one measure of burnout to another is only appropriate across measures that assess the same construct. A measure of emotional exhaustion (such as the MZSIB) cannot be crosswalked to derive an equivalent score on a metric of depersonalization.

## CONCLUSIONS

As US healthcare organizations are increasingly measuring physician burnout as an indicator of health system performance, there is a need to compare burnout outcomes across different assessments. Our findings allow healthcare organizations using the PFI or MZSIB to compare group-level scores to historical, regional, or national MBI scores (and vice-versa).

## Supplementary Information

ESM 1 (DOCX 80.7 kb)

## Abbreviations

IRT: Item response theory
MBI: Maslach Burnout Inventory-Human Services Survey for Medical Personnel
MBI-EE: Maslach Burnout Inventory-Human Services Survey for Medical Personnel Emotional Exhaustion scale
MBI-DP: Maslach Burnout Inventory-Human Services Survey for Medical Personnel Depersonalization scale
MZSIB: Mini-Z Single-Item Burnout scale
PFI: Stanford Professional Fulfillment Index
PFI-WE: Stanford Professional Fulfillment Index Work Exhaustion scale
PFI-ID: Stanford Professional Fulfillment Index Interpersonal Disengagement scale
