# Supplementary material for: Establishing Crosswalks Between Common Measures of Burnout in US Physicians
Source: J Gen Intern Med. 2021 Mar 31;37(4):777–84. doi: 10.1007/s11606-021-06661-4 (PMC8904666; doi:10.1007/s11606-021-06661-4)
Supplement: Supplementary file 1 — (DOCX 80.7 kb) [file 11606_2021_6661_MOESM1_ESM.docx]

**Supplemental Appendices – Brady et al. (2021) – Establishing Crosswalks between Common Measures of Burnout in US Physicians**

**Supplemental Appendix 1. Full Mini-Z Single-Item Burnout (MZSIB) Scale Response Options**

Response options and scoring for the MZSIB scale are as follows:

1 = “I enjoy my work. I have no symptoms of burnout.”

2 = “I am under stress, and don’t always have as much energy as I did, but don’t feel burned out.”

3 = “I am definitely burning out and have one or more symptoms of burnout, e.g., emotional exhaustion.”

4 = “The symptoms of burnout that I am experiencing won’t go away. I think about work frustrations a lot.”

5 = “I feel completely burned out. I am at the point where I may need to seek help.”

**Supplemental Appendix 2: Linking Assumption Assessment**

Prior to conducting linking analyses, we computed classic scale-level analyses, including Cronbach alpha coefficients and average inter-item correlations on each individual scale as well as on combined items in each item set (consisting of items from both the target and anchor scales that we planned to link). Next, we examined the degree to which the scales that we aimed to link met linking assumptions. These assumptions require that linked scales 1) are assessing essentially the same underlying construct, 2) are highly correlated, and 3) that standardized mean differences across subgroups (e.g., males vs females) are of a similar magnitude across target and anchor scales (subgroup invariance).^1,2^ We evaluated the first assumption qualitatively and quantitatively for each set of scales we aimed to link. First, two researchers independently rated the degree of item content overlap between each target scale (PFI-WE, PFI-ID, and MZSIB) and anchor scale (MBI-EE, MBI-DP) in a qualitative assessment. To evaluate the second assumption, we calculated correlations between each target and anchor measure. We considered a correlation coefficient $\geq$0.75 as adequate for linking. We evaluated the third assumption for each item set using the Root Mean Square Difference (RMSD) statistic across age, sex, and early/late responder groups. A RMSD value $\leq$8% was considered adequate for linking.^20^ The RMSD statistic assesses the degree to which standardized mean differences across subgroups (weighted by subgroup proportions) are of a similar magnitude across target and reference scales. ^20^

An additional assumption of item response theory (IRT) linking is that each item set is essentially unidimensional. We assessed essential unidimensionality in unidimensional and bifactor confirmatory factor analyses (CFAs) using weighted least squares mean- and variance-adjusted (WLSMV) estimation on polychoric correlations in the R *lavaan* package (version 0.6-3).^3^ An item set was considered unidimensional or essentially unidimensional if it showed adequate model fit to a unidimensional or bifactor CFA model, respectively. Adequate CFA model fit was defined as a scaled Root Mean Square Error Approximation (RMSEA) of $\leq$0.10, a scaled Tucker-Lewis Index (TLI) of $\geq$0.90, a scaled Comparative Fit Index (CFI) of $\geq$0.90, and a Standardized Root Mean Square Residual (SRMSR) of $\leq$0.08.^4-11^ Finally, we evaluated whether any major local dependence (LD) occurred in our IRT-based linking using Chen and Thissen (1997) LD X^2^ statistics.^12^ In alignment with Brady et al. (2020),{Brady KJS, (in press) #3565}^13^ item pairs that showed major LD violations were summed to form a single scale. This was performed to address LD violations while still allowing for all scale items to remain in the IRT model, which was critical for the purposes of linking existing scales.

Item sets were only eligible for linking if they met all these assumptions.

**Supplemental Appendix 3: Linking Methodology**

For each item set in item response theory (IRT) fixed calibration linking, items from the target scale were calibrated onto the metric of the anchor scale in an graded response IRT model where the item parameter estimates for the anchor scale were fixed to the estimates published in the Brady et al. (2020) study^13^ and the target items were freely estimated onto the anchor metric (latent mean and variance freely estimated). IRT models were estimated in the R *mirt* package.^14^ For each item set in equipercentile linking, we performed raw score to raw score equipercentile linking with loglinear pre-smoothing and bootstrapped standard errors in the R *equate* package.^15^ These IRT and equipercentile linking methods occurred in a five-fold cross validation process. That is, for each item set, the full sample was randomly split into five smaller samples (samples A, B, C, D, and E) with approximately even sample size. Next for each item set and linking method (IRT and equipercentile linking), we:

estimated/computed the linking model/function based on combined samples B, C, D, and E (*training dataset 1*), and evaluated the accuracy of the model in sample A (*test sample 1*);

estimated/computed the linking model/function based on combined samples A, C, D, and E (*training dataset 2*) and evaluated the accuracy of the model in sample B (test sample 2);

estimated/computed the linking model/function based on combined samples A, B, D, and E (*training dataset 3*) and evaluated the accuracy of the model in sample C (*test sample 3*);

estimated/computed the linking model/function based on combined samples A, B, C, and E (*training dataset 4*) and evaluated the accuracy of the model in sample D (*test sample 4*); and

estimated/computed the linking model/function based on combined samples A, B, C, and D (*training dataset 5*) and evaluated the accuracy of the model in sample E (*test sample 5*).

In IRT linking, after estimating the linking model based on each training dataset, we used the item parameter estimates for the target scale generated from the training dataset to obtain respondents' predicted MBI anchor scale scores. Predicted MBI anchor scores were computed using expected a posteriori (EAP) estimation for summed scores and converted to t-scores. Actual MBI anchor scores were also computed using EAP estimation for summed scores based on published MBI item parameter estimates^13^ and converted to t-scores. We then pooled actual and predicted t-scores from IRT linking test samples 1-5 and evaluated the accuracy of linking in the pooled sample for each item set.

In equipercentile linking, after computing a linking function based on each training dataset, we mapped each predicted raw score on the anchor scale to a scaled score using the expected test scores generated from IRT calibration of the MBI by Brady et al. (2020).^13^ We then pooled actual and predicted t-scores from equipercentile linking test samples 1-5 and evaluated the accuracy of linking in the pooled sample for each item set.

The final crosswalks from IRT linking were generated from each item set's full sample using the IRT estimation methods described above to place the target scale onto the metric of the MBI anchor scale in fixed calibration. We then used the item parameter estimates for the target scale to generate each item set's crosswalk mapping raw scores on the target scale to predicted z-scores on the MBI anchor metric using EAP estimation for summed scores. Z-scores on the MBI anchor measure were then converted to t-scores. Z-scores (and corresponding t-scores) were finally mapped to predicted raw scores on the MBI anchor scale using expected test score functions generated from Brady et al. (2020).^13^

The final crosswalks from equipercentile linking were generated from each item set's full sample using the equipercentile linking methods described above. We then mapped predicted raw scores on the MBI anchor scale to predicted t-scores using the expected test scores generated from Brady et al. (2020).^13^

**Supplemental Appendix 4: Computation of scale reliability across the MBI anchor scale t-score metric for scales linked to the same anchor metric**

For scales linked to the same MBI anchor metric, we computed each scale's reliability across MBI anchor scale t-scores using the test information functions generated from each final IRT model used to create each crosswalk. The reliability of the target and anchor scales linked to the same metric was then plotted in a single plot against MBI t-scores. The plots allow users to compare the reliability of scales linked to the same metric. The reliability implied by these plotted functions differs slightly from that which is implied by the standard errors in the crosswalks due to EAP estimation method used in generating the crosswalks.

**Supplemental Appendix 5: Linking Assessment Assumption Assessment Results**

Table 5.1. Classic Scale Analyses and Inter-Scale Correlations ^a^

| **Scale/Item Set** | **No. of items** | **Cronbach alpha** | **Average inter-item correlation** | **Inter-scale Pearson’s correlation between target and anchor scales** |
| --- | --- | --- | --- | --- |
| MBI Emotional Exhaustion Scale | 9 | 0.93 | 0.61 | --^b^ |
| PFI Work Exhaustion Scale | 4 | 0.87 | 0.63 | --^b^ |
| MBI Emotional Exhaustion scale and PFI Work Exhaustion scale (Item Set 1) | 13 | 0.95 | 0.58 | 0.80 |
| MBI Depersonalization scale | 5 | 0.84 | 0.52 | --^b^ |
| PFI Interpersonal Disengagement scale | 6 | 0.92 | 0.66 | --^b^ |
| MBI Depersonalization scale and PFI Interpersonal Disengagement scale (Item Set 2) | 11 | 0.93 | 0.55 | 0.76 |
| MBI Emotional Exhaustion scale and Mini-Z Single Item Burnout scale (Item Set 3) | 10 | 0.94 | 0.61 | 0.76 |

*^a^The Mini-Z single item burnout (MZSIB) scale is not listed because these statistics can only be calculated for multi-item measures. ^b^Not applicable.*

**Essential Unidimensionality Assessment**

All confirmatory factor analysis (CFA) models supported essential unidimensionality in either a unidimensional CFA or a bifactor CFA.

Table 5.2. Confirmatory Factor Analyses (CFA) Model Fit Indices – MBI Emotional Exhaustion scale and PFI Work Exhaustion scale (Item Set 1) ^a^

| Model | No. items | RMSEA | TLI | CFI | SRMSR |
| --- | --- | --- | --- | --- | --- |
| Unidimensional model of all MBI-EE and PFI-WE items (WLSMV estimation, polychoric correlations) | 13 | 0.120 | 0.982 | 0.985 | 0.064 |
| Final model: Bifactor model (MBI-EE items EE4 and EE8 combined in a single item. All items loading onto the general factor. All MBI-EE items on specific factor 1. All PFI-WE items loading onto specific factor 2) (R *psych* package, ML estimation, Pearson correlations) ^b^ | 12 | 0.083 | 0.951 | 0.969 | 0.025 |

*^a^ RMSEA, TLI, and CFA are scaled fit indices where WLSMV estimation is used. ^b^ Omega Hierarchical coefficient* $\omega_{H}$ *for Model 2 was 0.93, indicating a strong underlying general factor that explains correlations among all the MBI-EE and PFI-WE items.*

Table 5.3. Confirmatory Factor Analyses (CFA) Model Fit Indices – MBI Depersonalization scale and PFI Interpersonal Disengagement scale (Item Set 2) *^a^*

| Model | No. items | RMSEA | TLI | CFI | SRMSR |
| --- | --- | --- | --- | --- | --- |
| Model 1: Unidimensional model of all MBI-DP and PFI-ID items (WLSMV estimation, polychoric correlations) | 11 | 0.181 | 0.966 | 0.972 | 0.076 |
| Model 2 (Final model): Unidimensional model of all MBI-DP and PFI-ID items (PFI-ID items ID2 and ID6 combined into a single item and PFI-ID items ID4 and ID5 combined into a single item) (WLSMV estimation, polychoric correlations) | 9 | 0.102 | 0.980 | 0.985 | 0.056 |

*^a^ RMSEA, TLI, and CFA are scaled fit indices where WLSMV estimation is used.*

Table 5.4. Confirmatory Factor Analyses (CFA) Model Fit Indices – MBI Emotional Exhaustion scale and Mini-Z Single Item Burnout scale (Item Set 3) *^a^*

| Model | No. items | RMSEA | TLI | CFI | SRMSR |
| --- | --- | --- | --- | --- | --- |
| Model 1: Unidimensional model of all MBI-EE and Mini-Z SIB items (WLSMV, polychoric correlations) | 10 | 0.112 | 0.988 | 0.991 | 0.067 |
| Model 2 (Final model): Unidimensional model all MBI-EE and Mini-Z SIB items (EE items 4 and 8 combined into a single item) (WLSMV estimation, polychoric correlations) | 9 | 0.088 | 0.994 | 0.995 | 0.036 |

*^a^ RMSEA, TLI, and CFA are scaled fit indices where WLSMV estimation is used.*

**Subgroup Invariance Assessment**

Table 5.5. Root Mean Square Difference (RMSD) Statistics across Sex, Age, and Early/Late Responder Groups ^a^

| Item Set | **RMSD for sex groups (male; female)** | **RMSD for age groups (**$\boldsymbol{\leq}$**44 yrs;** $\boldsymbol{\geq}$**45 yrs)** | **RMSD for responder group (early responders; late responder)^b^** |
| --- | --- | --- | --- |
| Item set 1 (PFI-WE and MBI-EE) | 0.60% | 3.27% | 0.67% |
| Item set 2 (PFI-ID and MBI-DP) | 0.42% | 5.12% | 3.76% |
| Item set 3 (Mini-Z SIB and MBI-EE) | 0.57% | 0.24% | 0.86% |

*^a^ RMSD below <8% supports subgroup invariance. ^b^ Early responders were those that responded before 4/01/2019; late responders were those that responded on or after 4/01/2019. Response dates ranged from 2/20/2019 to 5/05/2019.*

**Supplemental Appendix 6. Descriptive Scale Statistics**

Table 6.1. Specialty-Level Descriptive Scale Statistics by Domain/Measure (n = 1,346) ^a^

|  | Emotional Exhaustion | | | | | | Depersonalization | | | | Burnout | | |
| --- | --- | --- | --- | --- | --- | --- | --- | --- | --- | --- | --- | --- | --- |
| Specialty | MBI-EE, mean (SD) | MBI-EE $\geq$27, n (%) | PFI-WE, mean (SD) | PFI-WE $\geq$7, n (%) | MZSIB, mean (SD) | MZSIB $\geq$3, n (%) | MBI-DP, mean (SD) | MBI-DP $\geq$10, n (%) | PFI-ID, mean (SD) | PFI-ID $\geq$9, n (%) | MBI (EE $\geq$27 and/or DP $\geq$10) , n (%) | PFI-BC ^b^, mean (SD) | PFI-BC ^b^ $\geq$14, n (%) |
| Anesthesiology (n = 97) | 22.15 (12.02) | 29 (29.9) | 6.39 (3.37) | 48 (49.5) | 2.52 (0.93) | 48 (49.5) | 8.05 (6.99) | 36 (37.1) | 7.31 (4.73) | 38 (39.2) | 41 (42.3) | 13.70 (7.49) | 46 (47.4) |
| Dermatology (n = 24) | 20.38 (12.56) | 7 (29.2) | 5.00 (3.16) | 7 (29.2) | 2.38 (1.01) | 7 (29.2) | 6.46 (6.45) | 6 (25.0) | 5.25 (4.41) | 6 (25.0) | 9 (37.5) | 10.25 (6.86) | 6 (25.0) |
| Emergency Medicine (n = 74) | 22.82 (9.92) | 22 (29.7) | 6.15 (2.85) | 29 (39.2) | 2.51 (0.83) | 36 (48.6) | 10.86 (6.76) | 42 (56.8) | 7.93 (4.27) | 34 (45.9) | 42 (56.8) | 14.08 (6.60) | 41 (55.4) |
| Family Medicine (n = 164) | 22.29 (12.81) | 61 (37.2) | 6.40 (3.66) | 80 (48.8) | 2.41 (0.96) | 70 (42.7) | 7.54 (6.18) | 47 (28.7) | 6.58 (5.03) | 58 (35.4) | 70 (42.7) | 12.98 (8.06) | 76 (46.3) |
| General Surgery (n = 62) | 21.79 (10.28) | 20 (32.3) | 6.48 (2.88) | 29 (46.8) | 2.58 (0.93) | 31 (50.0) | 8.90 (6.51) | 25 (40.3) | 7.31 (4.50) | 24 (38.7) | 28 (45.2) | 13.79 (6.67) | 34 (54.8) |
| General surgery subspecialty (n = 70) | 23.71 (12.24) | 29 (41.4) | 6.30 (3.06) | 32 (45.7) | 2.51 (0.79) | 31 (44.2) | 9.24 (6.53) | 25 (35.7) | 7.57 (4.95) | 30 (42.9) | 30 (42.9) | 13.87 (7.44) | 34 (48.6) |
| General Internal Medicine (n = 182) | 22.98 (12.82) | 75 (41.2) | 6.26 (3.49) | 81 (44.5) | 2.42 (0.88) | 79 (43.4) | 8.60 (6.68) | 75 (41.2) | 7.05 (4.82) | 68 (37.4) | 90 (49.5) | 13.32 (7.84) | 90 (49.5) |
| General Pediatrics (n = 90) | 20.83 (11.97) | 31 (34.4) | 6.23 (3.50) | 38 (42.2) | 2.41 (0.86) | 38 (42.2) | 6.29 (5.70) | 21 (23.3) | 5.96 (4.51) | 28 (31.1) | 36 (40.0) | 12.19 (7.27) | 34 (37.8) |
| Internal Medicine-subspecialty (n = 126) | 22.13 (11.86) | 46 (36.5) | 6.01 (3.41) | 58 (46.0) | 2.46 (0.96) | 59 (46.8) | 7.93 (5.87) | 46 (36.5) | 6.86 (4.22) | 51 (40.5) | 61 (48.4) | 12.87 (6.95) | 61 (48.4) |
| Neurology (n = 28) | 19.93 (14.78) | 9 (32.1) | 5.86 (4.90) | 11 (39.3) | 2.46 (1.17) | 12 (42.9) | 7.18 (6.90) | 8 (28.6) | 7.68 (6.82) | 11 (39.3) | 9 (32.1) | 13.54 (11.47) | 13 (46.4) |
| Obstetrics and gynecology (n = 96) | 22.22 (11.59) | 38 (40.0) | 6.26 (3.63) | 46 (47.9) | 2.61 (0.92) | 51 (53.1) | 7.06 (5.72) | 27 (28.1) | 6.18 (4.63) | 30 (31.3) | 44 (45.8) | 12.44 (7.71) | 43 (44.8) |
| Ophthalmology (n = 30) | 13.90 (8.30) | 2 (6.7) | 3.80 (2.43) | 4 (13.3) | 2.00 (0.59) | 5 (16.7) | 4.10 (5.14) | 2 (6.7) | 3.70 (3.90) | 3 (10.0) | 2 (6.7) | 7.50 (5.90) | 2 (6.7) |
| Other (n = 80) | 19.57 (12.37) | 23 (28.8) | 5.19 (3.24) | 27 (33.8) | 2.35 (0.98) | 29 (36.3) | 7.90 (6.90) | 31 (38.8) | 6.03 (4.95) | 24 (30.0) | 33 (41.3) | 11.21 (7.55) | 29 (36.3) |
| Pathology (n = 4) | 31.25 (11.18) | 2 (50.0) | 10.00 (4.08) | 3 (75.0) | 3.00 (0.82) | 3 (75.0) | 9.00 (8.37) | 2 (50.0) | 7.25 (6.08) | 2 (50.0) | 2 (50.0) | 17.25 (7.93) | 3 (75.0) |
| Pediatric subspecialty (n = 63) | 20.11 (12.06) | 20 (31.7) | 6.06 (3.51) | 29 (46.0) | 2.49 (0.88) | 31 (49.2) | 6.41 (4.90) | 14 (22.2) | 5.75 (4.28) | 13 (20.6) | 22 (34.9) | 11.81 (7.20) | 26 (41.3) |
| Physical medicine (n = 13) | 16.77 (12.20) | 3 (23.1) | 5.23 (3.32) | 4 (30.8) | 2.31 (0.95) | 4 (30.8) | 5.77 (5.88) | 3 (23.1) | 4.85 (4.00) | 3 (23.1) | 4 (30.8) | 10.08 (6.55) | 4 (30.1) |
| Psychiatry (n = 90) | 22.31 (12.31) | 29 (32.2) | 5.70 (3.62) | 36 (40.0) | 2.37 (0.91) | 35 (38.9) | 8.00 (7.04) | 24 (26.7) | 6.53 (5.18) | 31 (34.4) | 32 (35.6) | 12.23 (8.34) | 36 (40.0) |
| Radiology (n = 53) | 23.28 (13.22) | 23 (43.4) | 5.91 (3.71) | 20 (37.7) | 2.38 (1.02) | 20 (37.7) | 7.74 (5.94) | 24 (45.3) | 5.79 (4.53) | 16 (30.2) | 29 (54.7) | 11.70 (7.56) | 21 (39.6) |

^a^ Includes respondents with $\leq$1 missing item response for all scales. Cut-points presented are raw total scores on each scale. ^b^ PFI BC refers to the PFI Burnout Composite scale, which is scored as the total raw score from both the PFI-WE and PFI-ID scales.

**Supplemental Appendix 7: Results of Five-Fold Cross-Validation to Generate Accuracy Statistics for Each Linking Method and Overall Accuracy of Each Linking Method**

**Section 1. Results of Five-Fold Cross-Validation to Generate Accuracy Statistics for Each Linking Method**

Table 7.1. Five-Fold Cross Validation: Item Set 1 – PFI-WE Scale Linked to MBI-EE scale

| **Linking method/ sample** | **n** | **Correlation between predicted and actual anchor metric t-scores** | **Mean Difference between predicted and actual anchor metric t-score** | **SD of difference between predicted and actual anchor metric t-scores** |
| --- | --- | --- | --- | --- |
| IRT FIXED CALIBRATION | | | | |
| Test sample A | 269 | 0.79 | -0.15 | 5.25 |
| Test sample B | 267 | 0.76 | -0.02 | 5.68 |
| Test sample C | 265 | 0.82 | -0.53 | 5.38 |
| Test sample D | 269 | 0.81 | 0.56 | 5.35 |
| Test sample E | 267 | 0.79 | 0.83 | 5.39 |
| EQUIPERCENTILE LINKING | | | | |
| Test sample A | 269 | 0.79 | -0.60 | 5.42 |
| Test sample B | 267 | 0.76 | -0.40 | 5.96 |
| Test sample C | 265 | 0.82 | -0.91 | 5.72 |
| Test sample D | 269 | 0.82 | 0.18 | 5.49 |
| Test sample E | 267 | 0.78 | 0.52 | 5.84 |

Table 7.2. Five-Fold Cross Validation: Item Set 2 – PFI ID Scale Linked to MBI-DP Scale

| **Linking method/ sample** | **n** | **Correlation between predicted and actual anchor metric t-scores** | **Mean Difference between predicted and actual anchor metric t-score** | **SD of difference between predicted and actual anchor metric t-scores** |
| --- | --- | --- | --- | --- |
| IRT FIXED CALIBRATION | | | | |
| Test sample A | 269 | 0.77 | 0.50 | 5.86 |
| Test sample B | 268 | 0.77 | 0.60 | 5.92 |
| Test sample C | 267 | 0.76 | 0.54 | 6.14 |
| Test sample D | 267 | 0.77 | 0.23 | 6.08 |
| Test sample E | 266 | 0.71 | 0.52 | 6.56 |
| EQUIPERCENTILE LINKING | | | | |
| Test sample A | 269 | 0.72 | -2.19 | 9.86 |
| Test sample B | 268 | 0.73 | -1.99 | 9.98 |
| Test sample C | 267 | 0.74 | -1.99 | 10.03 |
| Test sample D | 267 | 0.73 | -2.93 | 10.57 |
| Test sample E | 266 | 0.67 | -2.63 | 10.81 |

Table 7.3. Five-Fold Cross Validation: Item Set 3 –MZSIB Scale Linked to MBI-EE Scale

| **Linking method/ sample** | **n** | **Correlation between predicted and actual anchor metric t-scores** | **Mean Difference between predicted and actual anchor metric t-score** | **SD of difference between predicted and actual anchor metric t-scores** |
| --- | --- | --- | --- | --- |
| **IRT FIXED CALIBRATION** | | | | |
| Test sample A | 269 | 0.77 | 0.06 | 5.40 |
| Test sample B | 267 | 0.75 | 0.76 | 5.70 |
| Test sample C | 268 | 0.72 | 0.16 | 6.18 |
| Test sample D | 269 | 0.78 | 1.81 | 5.61 |
| Test sample E | 268 | 0.76 | 1.40 | 5.57 |
| **EQUIPERCENTILE LINKING** | | | | |
| Test sample A | 269 | 0.77 | -0.88 | 5.54 |
| Test sample B | 267 | 0.74 | -0.30 | 5.90 |
| Test sample C | 268 | 0.72 | -0.95 | 6.36 |
| Test sample D | 269 | 0.77 | 0.93 | 6.03 |
| Test sample E | 268 | 0.76 | 0.39 | 5.71 |

**Section 2. Overall Accuracy of IRT and Equipercentile Linking Methods**

Evaluation of the correlations, mean difference, SD of the difference between predicted and actual anchor metric t-scores produced from five-fold cross-validation demonstrated that IRT linking outperformed equipercentile linking (Supplemental Appendix Table 7.4). Compared to equipercentile linking, IRT linking produced comparable or higher correlations between predicted and actual anchor t-scores in all item sets and predicted anchor t-scores, with smaller mean differences and SD of differences in most or all item sets. Anchor scale t-scores predicted by each method for Item Sets 1-3 correlated at 0.998, 0.978, 0.997, respectively, supporting the robustness of our linking analyses.

**Table 7.4 Overall Accuracy of IRT and Equipercentile Linking Methods: Correlation, mean difference, and standard deviation of difference between predicted and actual anchor metric t-scores for each item set ^a^**

| **Item Set/Linking Method** | **n** | **Correlation between predicted and actual anchor metric t-scores** | **Mean Difference between predicted and actual anchor metric t-score** | **SD of difference between predicted and actual anchor metric t-scores** |  |
| --- | --- | --- | --- | --- | --- |
| **ITEM SET 1: 4-item PFI Work Exhaustion (PFI-WE) subscale (target scale) linked to 9-item MBI Emotional Exhaustion (MBI-EE) subscale (anchor scale)** | | | | | |
| Item response theory ^a^ | 1337 | 0.79 | 0.14 | 5.43 |  |
| Equipercentile linking | 1337 | 0.79 | -0.24 | 5.70 |  |
| **ITEM SET 2: 6-item PFI Interpersonal Disengagement (PFI-ID) subscale (target scale) linked to 5-item MBI Depersonalization (MBI-DP) subscale (anchor scale)** | | | | | |
| Item response theory ^a^ | 1337 | 0.76 | 0.48 | 6.11 |  |
| Equipercentile linking | 1337 | 0.72 | -2.34 | 10.25 |  |
| **ITEM SET 3 Mini-Z Single-Item Burnout (MZSIB) scale (target scale) linked to MBI-EE subscale (anchor scale)** | | | | | |
| Item response theory ^a^ | 1341 | 0.75 | 0.84 | 5.73 |  |
| Equipercentile linking | 1341 | 0.75 | -0.16 | 5.95 |  |

*^a^ Item response theory linking used fixed-calibration linking, whereby the metric of the anchor scale was fixed to the metric derived in a prior IRT analysis of 2014 MBI response data obtained from a national sample of US physicians.^13^ Accuracy statistics were calculated from predicted and observed anchor scale scores generated from five-fold cross validation.*

**Supplemental Appendix 8. IRT (Fixed Calibration) Item Parameter Estimates for Each Target Scale on the MBI Metric**

Tables 8.1-8.3 present the transformed item parameter estimates for each item set's target scale produced from each final fixed calibration IRT model.

Table 8.1. Transformed Item Parameter Estimates (Fixed Parameter Calibration) – Stanford Professional Fulfillment Index Work Exhaustion (PFI-WE) Scale Linked to MBI Emotional Exhaustion (MBI-EE) Metric (Item Set 1) ^a^

| **Item** | **Slope** | **TP1** | **TP2** | **TP3** | **TP4** |
| --- | --- | --- | --- | --- | --- |
| PFI-WE1 | 2.84 | -1.14 | -0.19 | 0.78 | 1.67 |
| PFI-WE2 | 2.52 | -1.39 | -0.43 | 0.62 | 1.87 |
| PFI-WE3 | 2.54 | -1.33 | -0.19 | 0.83 | 1.78 |
| PFI-WE4 | 3.45 | -1.2 | -0.25 | 0.6 | 1.48 |

^a^ *TP= threshold parameter*

Table 8.2. Transformed Item Parameter Estimates (Fixed Parameter Calibration) – Stanford Professional Fulfillment Index Interpersonal Disengagement (PFI-ID) Scale Linked to MBI Depersonalization Scale (MBI-DP) (Item Set 2)

| **Item** | **Slope** | **TP1** | **TP2** | **TP3** | **TP4** | **TP5** | **TP6** | **TP7** | **TP8** |
| --- | --- | --- | --- | --- | --- | --- | --- | --- | --- |
| *PFI-ID1* | 3.82 | -0.39 | 0.60 | 1.56 | 2.47 | -- | -- | -- | -- |
| *PFI-ID3* | 3.80 | -0.52 | 0.49 | 1.49 | 2.39 | -- | -- | -- | -- |
| *PFI-ID4ID5^b^* | 3.54 | -0.57 | -0.38 | 0.44 | 0.70 | 1.42 | 1.66 | 2.39 | 2.64 |
| *PFI-ID2ID6^b^* | 2.62 | -0.78 | -0.38 | 0.33 | 0.72 | 1.35 | 1.7 | 2.37 | 2.88 |

^a^ *TP= threshold parameter; PFI items ID4 and ID5 were combined into a single item (PFI-ID4ID5) to meet the local independence assumption of IRT. PFI items ID2 and ID6 were combined into a single item (PFI-ID2ID6) to meet the local independence assumption of IRT.*

Table 8.3. Transformed Item Parameter Estimates (Fixed Parameter Calibration) – Mini-Z Single Item Burnout (MZSIB) Scale Linked to MBI Emotional Exhaustion Metric (Item Set 3) ^a^

| **Item** | **Slope** | **TP1** | **TP2** | **TP3** | **TP4** |
| --- | --- | --- | --- | --- | --- |
| MZSIB Scale | 3.31 | -1.33 | -0.14 | 0.77 | 2.14 |

^a^ *TP= threshold parameter*

**Supplemental Appendix 9. Crosswalks produced from equipercentile linking mapping raw scores from the PFI and Mini-Z SIB to corresponding predicted MBI t-scores and raw scores**

Tables 9.1-9.3 present the crosswalks produced from the final equipercentile linking functions for each item set. We present these crosswalks for completeness. We recommend the use of the IRT-based crosswalks presented in Table 3 of the main manuscript, as IRT linking was generally more accurate than the equipercentile linking in our analyses.

Table 9.1. PFI Work Exhaustion (PFI-WE) Scale (Target Scale) Linked to MBI Emotional Exhaustion (MBI-EE) Scale (Anchor Scale) - Based on Equipercentile Linking

| PFI-WE raw (total) score | Predicted MBI-EE raw (total) score (Bootstrapped SE) | Predicted MBI-EE t-score |
| --- | --- | --- |
| 0 | 2.20 (0.35) | 29.42 |
| 1 | 5.37 (0.47) | 34.36 |
| 2 | 7.97 (0.48) | 37.22 |
| 3 | 10.62 (0.50) | 39.59 |
| 4 | 13.54 (0.57) | 41.87 |
| 5 | 16.88 (0.65) | 44.24 |
| 6 | 20.69 (0.73) | 46.72 |
| 7 | 24.90 (0.76) | 49.31 |
| 8 | 29.28 (0.73) | 51.97 |
| 9 | 33.47 (0.68) | 54.69 |
| 10 | 37.22 (0.64) | 57.39 |
| 11 | 40.39 (0.64) | 59.90 |
| 12 | 43.03 (0.70) | 62.16 |
| 13 | 45.21 (0.81) | 64.31 |
| 14 | 47.10 (0.92) | 66.64 |
| 15 | 48.97 (0.99) | 69.83 |
| 16 | 51.52 (0.85) | 77.11 |

Table 9.2. PFI Interpersonal Disengagement (PFI-ID) Scale (Target Scale) Linked to MBI Depersonalization (MBI-DP) Scale (Anchor Scale) - Based on Equipercentile Linking

| PFI-ID Raw (Total) Score | Predicted MBI-DP Raw (Total) Score (Bootstrapped SE) | Predicted MBI-DP t-score |
| --- | --- | --- |
| 0 | 0.25 (0.07) | 17.79 |
| 1 | 1.45 (0.15) | 36.47 |
| 2 | 2.32 (0.17) | 40.69 |
| 3 | 3.09 (0.19) | 43.04 |
| 4 | 3.86 (0.21) | 44.88 |
| 5 | 4.70 (0.23) | 46.61 |
| 6 | 5.67 (0.26) | 48.39 |
| 7 | 6.84 (0.29) | 50.29 |
| 8 | 8.24 (0.33) | 52.29 |
| 9 | 9.91 (0.38) | 54.43 |
| 10 | 11.83 (0.44) | 56.72 |
| 11 | 13.93 (0.49) | 59.14 |
| 12 | 16.05 (0.53) | 61.61 |
| 13 | 18.02 (0.55) | 64.04 |
| 14 | 19.76 (0.56) | 66.40 |
| 15 | 21.24 (0.58) | 68.59 |
| 16 | 22.44 (0.61) | 70.52 |
| 17 | 23.42 (0.65) | 72.26 |
| 18 | 24.21 (0.69) | 73.84 |
| 19 | 24.82 (0.73) | 75.22 |
| 20 | 25.30 (0.77) | 76.42 |
| 21 | 25.73 (0.81) | 77.59 |
| 22 | 26.19 (0.84) | 78.96 |
| 23 | 26.78 (0.86) | 80.92 |
| 24 | 28.15 (0.80) | 86.69 |

Table 9.3. Mini-Z Single-Item Burnout (MZSIB) Scale (Target Scale) Linked to MBI Emotional Exhaustion (MBI-EE) Scale (Anchor Scale) - Based on Equipercentile Linking

| Mini-Z SIB Raw (Total) Score | Predicted MBI-EE Raw (Total) Score (Bootstrapped SE) | Predicted MBI-EE t-score |
| --- | --- | --- |
| 1 | 4.98 (0.35) | 33.86 |
| 2 | 15.45 (0.46) | 43.25 |
| 3 | 29.08 (0.64) | 51.85 |
| 4 | 41.05 (0.61) | 60.45 |
| 5 | 51.26 (0.84) | 76.11 |

**Supplemental Appendix 10. Example of How to Use Crosswalks in Table 3 of the Main Manuscript**

The following example demonstrates how to use the (IRT-based) crosswalks in Table 3 of the main manuscript to identify emotional exhaustion and depersonalization rates on the MBI-EE and MBI-DP scales using group-level PFI-WE and PFI-ID data, respectively. PFI data for this example were obtained from a cross-sectional sample of 877 physicians who responded to the 2017 Stanford Physician Wellness Survey. For the purposes of this example, we will assume that all respondents had complete responses on all PFI-WE and PFI-ID items. Using the item-level PFI response data from this survey, we generated the frequency tables presented in Tables 10.1 and 10.2. These tables describe the *number* and *percent* of survey respondents who scored each possible raw (total) score point on the PFI-WE and PFI-ID scales, respectively. For example, from Table 10.1, we know that 48 physicians (48/877, or 5.47%) scored a raw score of 0 on the PFI-WE scale, 52 physicians (52/877, or 5.93%) scored a raw score of 1, 45 physicians (45/877, or 5.13%), etc. In this example, an investigator wants to compare the emotional exhaustion and depersonalization rates at Stanford (assessed using the PFI) to national benchmarks reported in Shanafelt et al. (2019),^16^ which are assessed using the MBI. Using the crosswalks in Table 3 of the main manuscript, we can see that a raw (total) score of $\geq$7 on the PFI-WE scale is the closest equivalent score to the MBI-EE raw (total) score cut-point of $\geq$27; and a raw score of $\geq$9 on the PFI-ID scale is the closest equivalent score to the MBI-DP raw (total) score cut-point of $\geq$10. Therefore, to calculate the percent of physicians who are emotionally exhausted and depersonalized on the MBI-EE and MBI-DP scales, respectively, we would sum the number of physicians in Table 10.1 with a PFI-WE raw score $\geq$7 and the number of physicians in Table 10.2 with a PFI-ID raw score $\geq$9. Summing the number of physicians who scored $\geq$7 on the PFI-WE equals 319 out of 877 physicians, or 36.4% of physicians that are (predicted to be) emotionally exhausted on the MBI-EE metric. Summing the number of physicians who scored $\geq$9 on the PFI-ID equals 189 out of 877 physicians, or 21.6% of physicians that are (predicted to be) depersonalized exhausted on the MBI-DP metric. Compared to the 2019 national rates of emotional exhaustion and depersonalization of 38.7% and 27.3%, respectively, reported in Shanafelt et al. (2019), there are lower rates of emotional exhaustion and depersonalization in this sample.

**Table 10.1 Number and Percent of Survey Respondents Scoring Each PFI-WE Score (n=877)**

| PFI-WE raw (total) score | Number of respondents with this score | Percent of respondents with this score |
| --- | --- | --- |
| 0 | 48 | 5.47% |
| 1 | 52 | 5.93% |
| 2 | 45 | 5.13% |
| 3 | 79 | 9.01% |
| 4 | 128 | 14.6% |
| 5 | 107 | 12.2% |
| 6 | 99 | 11.29% |
| 7 | 75 | 8.55% |
| 8 | 76 | 8.67% |
| 9 | 48 | 5.47% |
| 10 | 25 | 2.85% |
| 11 | 29 | 3.31% |
| 12 | 30 | 3.42% |
| 13 | 12 | 1.37% |
| 14 | 10 | 1.14% |
| 15 | 6 | 0.68% |
| 16 | 8 | 0.91% |
| Total | 877 | 100% |

**PFI interpersonal disengagement (n=876, missing respondent=1)**

| Interpersonal disengagement score point | Number at this score point | Percent at this score point |
| --- | --- | --- |
| 0 | 186 | 21.23% |
| 1 | 44 | 5.02% |
| 2 | 53 | 6.05% |
| 3 | 56 | 6.39% |
| 4 | 41 | 4.68% |
| 5 | 53 | 6.05% |
| 6 | 159 | 18.15% |
| 7 | 64 | 7.31% |
| 8 | 31 | 3.54% |
| 9 | 50 | 5.71% |
| 10 | 33 | 3.77% |
| 11 | 14 | 1.6% |
| 12 | 35 | 4% |
| 13 | 12 | 1.37% |
| 14 | 9 | 1.03% |
| 15 | 8 | 0.91% |
| 16 | 3 | 0.34% |
| 17 | 2 | 0.23% |
| 18 | 10 | 1.14% |
| 19 | 3 | 0.34% |
| 20 | 2 | 0.23% |
| 21 | 1 | 0.11% |
| 22 | 1 | 0.11% |
| 23 | 2 | 0.23% |
| 24 | 4 | 0.46% |
| Total | 876 | 100% |

**References**

1. Dorans NJ, Holland PW. Population invariance and the equatability of tests: Basic theory and the linear case. *J Educ Meas.* 2000;37(4):281-306.

2. Kolen MJ, Brennan RL. Test equating, scaling, and linking: Methods and practices. Springer Science & Business Media; 2014.

3. Rosseel Y. lavaan: An R Package for Structural Equation Modeling. *Journal of Statistical Software.* 2012;48(2):1-36.

4. Hu Lt, Bentler PM. Cutoff criteria for fit indexes in covariance structure analysis: Conventional criteria versus new alternatives. *Structural equation modeling: a multidisciplinary journal.* 1999;6(1):1-55.

5. Steiger JH. Structural model evaluation and modification: An interval estimation approach. *Multivariate behavioral research.* 1990;25(2):173-180.

6. Chen F, Curran PJ, Bollen KA, Kirby J, Paxton P. An empirical evaluation of the use of fixed cutoff points in RMSEA test statistic in structural equation models. *Sociological methods & research.* 2008;36(4):462-494.

7. Muthén BO. Robust inference using weighted least squares and quadratic estimating equations in latent variable modeling with categorical and continuous outcomes. *Psychometrika.* 1997.

8. Satorra A, Bentler PM. A scaled difference chi-square test statistic for moment structure analysis. *Psychometrika.* 2001;66(4):507-514.

9. Xia Y. Investigating the chi-square-based model-fit indexes for WLSMV and ULSMV estimators. 2016.

10. HealthMeasures. PROMIS Instrument Development and Scientific Standards Version 2.0. 2013. Available at: https://www.healthmeasures.net/images/PROMIS/PROMISStandards_Vers2.0_Final.pdf. Accessed June 19, 2020.

11. Reeve BB, Hays RD, Bjorner JB, et al. Psychometric evaluation and calibration of health-related quality of life item banks: plans for the Patient-Reported Outcomes Measurement Information System (PROMIS). *Medical care.* 2007;45(5):S22-S31.

12. Thissen D, Pommerich M, Billeaud K, Williams VS. Item response theory for scores on tests including polytomous items with ordered responses. *Appl Psych Meas.* 1995;19(1):39-49.

13. Brady KJS NP, Sheldrick RC, Trockel MT, Shanafelt T, Rowe SG, Schneider JI, Kazis LE. Describing the Emotional Exhaustion, Depersonalization, and Low Personal Accomplishment Symptoms Associated with Maslach Burnout Inventory Subscale Scores in US Physicians. *Journal of Patient Reported Outcomes.* 2020;4(1):42.

14. Chalmers P. mirt: A Multidimensional Item Response Theory Package for the R Environment. *Journal of Statistical Software.* 2012;48(6):1-29.

15. Albano AD. equate: An R package for observed-score linking and equating. *Journal of Statistical Software.* 2016;74(8):1-36.

16. Shanafelt TD, West CP, Sinsky C, et al. Changes in Burnout and Satisfaction With Work-Life Integration in Physicians and the General US Working Population Between 2011 and 2017. *Mayo Clinic Proceedings*. 2019;90(12):1600-1613.
